# Combined metabolomics and proteomics to reveal beneficial mechanisms of *Dendrobium fimbriatum* against gastric mucosal injury

**DOI:** 10.3389/fphar.2022.948987

**Published:** 2022-08-30

**Authors:** Jing Sun, Peng-Fei Liu, Jia-Ni Liu, Cong Lu, Li-Tao Tong, Yong-Quan Wang, Jia-Meng Liu, Bei Fan, Feng-Zhong Wang

**Affiliations:** ^1^ Risk Assessment Laboratory of Agricultural Products Processing Quality and Safety, Key Laboratory of Agricultural Products Quality and Safety Collection, Storage and Transportation Control (Ministry of Agriculture and Rural Affairs), Institute of Agricultural Products Processing, Chinese Academy of Agricultural Sciences, Beijing, China; ^2^ Agricultural Product Storage and Processing Institute, Gansu Academy of Agricultural Sciences, Lanzhou, China; ^3^ School of Pharmacy, Hunan University of Chinese Medicine, Changsha, China

**Keywords:** *Dendrobium fimbriatum*, gastric mucosal injury, metabolomics, proteomics, pathway

## Abstract

As a dietary and medicinal plant, *Dendrobium fimbriatum* (DF) is widely utilized in China for improving stomach disease for centuries. However, the underlying mechanisms against gastric mucosal injury have not been fully disclosed. Here, metabolomics and proteomics were integrated to clarify the in-depth molecular mechanisms using cyclophosphamide-induced gastric mucosal injury model in mice. As a result, three metabolic pathways, such as creatine metabolism, arginine and proline metabolism, and pyrimidine metabolism were hit contributing to DF protective benefits. Additionally, γ-L-glutamyl-putrescine, cytosine, and thymine might be the eligible biomarkers to reflect gastric mucosal injury tatus, and DF anti-gastric mucosal injury effects were mediated by the so-called target proteins such as Ckm, Arg1, Ctps2, Pycr3, and Cmpk2. This finding provided meaningful information for the molecular mechanisms of DF and also offered a promising strategy to clarify the therapeutic mechanisms of functional foods.

## 1 Introduction

*Dendrobium* belongs to the Orchidaceae family and has been utilized as a precious tonic hygienic food in China for centuries. There are 76 species of *Dendrobium* plants distributed in China. Due to its sweet taste, *Dendrobium* is frequently used as complemental material when preparing tea, soup, and pastries. In addition, it is widely employed as a health supplement in high-quality food products or associated with herbal medicines for the treatment of various diseases, attributing to the health benefit such as nourishing the stomach, promoting secretion of saliva and reducing fever (Zhao et al., 2016; Zeng et al., 2017; Zhang et al., 2018). In recent years, many studies have demonstrated that extracts of various *Dendrobium* plants were effective when being implemented for the treatment of stomach diseases that may dramatically affect the life quality (Min et al., 2009; Takeuchi et al., 2014; Zhao et al., 2019).

Polysaccharides are always deemed as components being responsible for the therapeutical spectrum of *Dendrobium* that exerts gastric mucosal protection effects by down-regulating the ratio of Bax/Bcl2 in gastric mucosa, thus inhibiting the recurrence of gastric ulcer (Lu et al., 2008; Zhang et al., 2012; Zeng et al., 2017). However, our previous *in vitro* assays demonstrated that in comparison of polysaccharides, the phenolic compounds of *Dendrobium* exhibited better gastric mucosal protective activities, especially for the variety of *D. fimbriatum* (DF), and the primary constituents of DF have been clarified, such as bibenzyls, phenanthrenes, flavones, etc. (Liu et al., 2021). The chemical components and biological activities of different varieties of *Dendrobium* are quite different. For instance, *D. officinale* is rich of polysaccharides, which has good hypoglycemic effect (Qu et al., 2021). *D. devonianum* contains many dibenzyl compounds, which have good anti-tumor activity (Wu L. et al., 2019). There are many alkaloids, e.g., dendrobine in *D. nobile,* and those alkaloids have good effect for the treatment of Parkinson’s disease (Li et al., 2021). Glycosides and dibenzyl dimers are the main chemical families in DF, and own certain anti-tumor, anti-oxidation and liver protection effects. However, the quality indicators of DF are not specified in Chinese pharmacopoeia. DF is usually discarded, and little is known for the in-depth mechanisms for gastric mucosal protection.

In our previous studies (Liu et al., 2021), the chemical compositions of seven different species of *Dendrobium* have been profiled, including *D. denneanum*, *D. fimbriatum*, *D. nobile*, *D. thyrsiflorum*, *D. chrysotoxum*, *D. officinale*, and *D. devonianum*, and the results showed that the chemical components of *D. chrysotoxum* and DF were quite different from others. Furthermore, GES-1 cell treated by ethanol was employed to evaluate the protective effect of different species, and as a result, DF exhibited the greatest effect. Thus, in order to clarify deeply the protective mechanisms of DF on gastric mucosa, cyclophosphamide-induced gastric mucosa injury model in mice is used, and metabolomics and proteomics are employed in combination to explore the target and pathway of DF on gastric mucosa injury. The obtained findings are also envisioned to lay a foundation for the formulation of quality standards and further development and exploitation of DF.

Multi-omics has been recognized as a good pipeline towards exploring the mechanism of traditional Chinese medicine, through integrating including metabolomics, proteomics, transcriptomics, and genomics. Untargeted metabolomics is designed to measure as many metabolites as desired to obtain patterns or fingerprints of predefined samples (Henning et al., 2014). It is an important comparative tool for researches on a large number of unspecified metabolites. Proteomics is advantageous at unveiling unanswered questions from metabolomics analysis because it provides a powerful approach for the screening of variations in protein levels and post-translational modifications associated with the disease. It can identify several potential therapeutic targets and disease-related biomarkers (Wang et al., 2017). Multi-omics technology has certain applications regarding elucidating the physiological mechanisms and pharmacological action mechanisms of *Dendrobium*, especially for metabolomics. For example, Gu et al. (2022) used metabonomics to reveal the differences among *Dendrobium* varieties, Yuan et al. clarified the synthesis mechanism of flavonoids in *Dendrobium* by metabolomics and transcriptomics techniques (Yuan et al., 2022), and Zheng et al. (2017) identified biomarkers related to diseases through metabolomics and proteomics techniques. However, there is still lack of in-depth research on the mechanism of gastric mucosal protection of *Dendrobium spp*., especially DF. Therefore, in current study, the target and metabolic pathway of DF are studied *in vivo* according to employing both metabolomics and proteomic.

The overall aim of the present study was to capture the acute metabolic response after oral administration of DF ethanol extracts (DF) in mice. The gastric mucosal injury model was established by injecting CTX, and the metabolite and protein profiles of stomach tissues were measured by UHPLC-Q Exactive-MS. Finally, we attempted to address the issue by utilizing metabolomics and proteomics to explore the mechanisms of DF on mice model, which provides theoretical support for the development of *Dendrobium*-derived functional food.

## 2 Materials and methods

### 2.1 Reagents and materials

DF was purchased from Longmu *Dendrobium* Department of Longshan County, Longshan town (Yunnan, China), and authenticated by Prof. Jun Li following the features described in Chinese Pharmacopoeia (2020 edition). Detection kits for the measurement of malondialdehyde (MDA), 5-hydroxytryptamine (5-HT), prostaglandin E2 (PGE2), nitric oxide (NO), and nuclear factor kappa-B (NF-κB) were purchased from OriGene. Stanining kit (HE) was purchased from Ying Ze Bio (Beijing, China). CTX and Omeprazole (OPZ) were purchased from Solarbio (Beijing, China). Methanol, formic acid, and acetonitrile of LC-MS grade were purchased from Fisher Scientific Inc. (Geel, Belgium). Ultra-pure water was prepared by a Milli-Q integral water purification system.

### 2.2 Animals

Mice were supplied by Laboratory Animal Center, Institute for Drug Control, Ministry of Health. SPF Experimental Animal Research Center for help with mice experiments [SYXK (Beijing) 2014-0003]. Forty male Kunming mice weighing between 18 g and 22 g were used in this experiment. All animals were kept in the laboratory at room temperature (23°C) and fed in groups during the pretrial period. All animals were handled and cared according to the Guiding Principles of the Care and Use of Animals.

### 2.3 Preparation of *Dendrobium fimbriatum*

Thin slices of DF were extracted with 95% ethanol and 75% ethanol for an hour, successively. The resultant ethanol extract was combined, and freeze-dried, resulting in 311.6 g of a brown, powdery, crude ethanol extract of DF.

### 2.4 Study groups and diets

All mice were randomly divided into four groups (*n* = 10 for each), such as normal control group, model group, OPZ group (4 mg/kg), and DF group (400 mg/kg). All drugs and compounds in treated groups were administered once per day for ten consecutive days through gavage. Meanwhile, the control and model groups were treated with saline. Additionally, mice in all groups, except the control group, were given CTX (80 mg/kg) on the 4th, 6th, and 8th days, respectively. Body weight of each animal was recorded weekly. All animals were sacrificed at 10 days after of treatment. The stomach was immediately isolated, opened along the greater curvature, and washed with saline to remove blood clots and gastric juices. It was then divided into four parts for histological, ELISA, metabolomics, and proteomics analyses.

### 2.5 Histological analysis

Specimens with gastric mucosal lesions were fixed in a 10% formaldehyde solution, dehydrated using xylene and graded alcohols, then embedded in paraffin. Paraffin sections were prepared, cut at a thickness of 4 mm, and stained with hematoxylin and eosin according to standard procedures. Structural changes in gastric mucosa were visualized by a light microscope.

### 2.6 Assessment of cytokines expressions in stomach tissues

The tissue samples were thawed and stored at 2°C–8°C. After adding PBS to pH 7.4, the samples were thoroughly homogenized, and the supernatant was collected after centrifugation at 3,000 r/min for 20 min. After the pretreatment of the tissue samples, the level of MDA, 5-HT, PGE2, NO, and NF-κB were measured using commercial ELISA kits, according to the manufacturer’s instructions.

### 2.7 Metabolomics analysis based on UHPLC-Q Exactive-MS

Preserved stomach samples (−80°C) were thawed at room temperature for further analysis. 50°mg samples were placed into 2 ml centrifuge tubes along with steel balls (6 mm in diameter, one per tube). 400°μl of extract (methanol: water = 4:1) and 20 μl of the internal standard (0.3 mg/ml, L-2-chloro-phenylalanine, dissolved with acetonitrile) were then added to each centrifuge tube 6 min before grinding (frozen tissue grinder, −10°C, 50 Hz). The mixture was afterward ultrasonicated at 5°C for 30 min and left to rest at −20°C for 30°min. The samples were finally centrifuged at 13,000 ×°g (4°C). The resulting supernatants were pipetted into sample injections for analysis. Ten samples were randomly selected from each group for the metabolomics research. Chromatographic analysis was also performed using Vanquish Horizon UHPLC system (Waters Corporation, Milford, United States). A 2 μl aliquot of sample solution was injected into a BEH C_18_ column (2.1°mm × 100 mm, 1.8 μm) at a flow rate of 0.4 ml/min (40°C). The mobile phase was 0.1% formic acid in water (A) and 0.1% formic acid in acetonitrile/2-Propanol (1/1) (B). The gradient elution was 0–3°min, 95%–80% A, 3–9°min, 80%–5% A, 9–13°min, 5% A, 13–13.1°min, 5%–95% A, and 13.1–16°min, 95% A. Additionally, a QC sample was used for parameter optimization of the UHPLC-Q Exactive-MS (Waters Corporation, Milford, United States) since it contained most of the sample-related information.

A mass spectrometry analysis was performed using a Q Exactive system. The optimal analysis conditions were as follows: Positive and negative modes; Auxiliary gas temperature of 400°C; Collision energy range of 20–40 eV. Mass spectra were recorded across a range of 70–1050 Da.

### 2.8 Multivariate statistical analysis and differential metabolites identification

Raw data were imported into Progenesis QI software (Waters Corporation, Milford, United States). Peak detection, alignment, and identification were successively carried out using Progenesis QI. Finally, database retrieval on HMDB, Metlin, as well as other databases was performed to establish the required parameters in the early stages of our research. The formula prediction errors were within ±5 ppm, the pre-processed data was carried out by *t*-test in combination with multivariate analysis to screen out differential metabolites. In the *S*-plot chart, the metabolites that VIP >1 and *p*-value < 0.05 were selected and imported into Progenesis QI, and compare the databases to identify metabolites.

### 2.9 SWATH proteomic analysis

#### 2.9.1 Creation of a spectral library

Protein solutions, quality inspections, and peptide sample preparations were carried out according to previous methods (Barber and Harris, 1994; Luo et al., 2019; Wu W. et al., 2019). Equal amounts of enzymatically digested peptide samples were mixed and concentrated by vacuum centrifugation before reconstitution with UPLC loading buffer. A high pH liquid phase separation and chromatographic analysis were performed using ACQUITY UPLC BEH C_18_ column (Waters Corporation, United States) and Vanquish Flex UHPLC (Thermo Scientific, United States). The pH value was adjusted to 10 with ammonia in 2% acetonitrile (A) and ammonia in 80% acetonitrile (B) during the mobile phase. The gradient elution was programmed as follows: 0–16 min, 0% B; 16–17 min, 0%–3.8% B; 17–34 min, 3.8%–24% B; 34–37 min, 24%–30% B; 37–38 min, 30%–43% B; 38–39 min, 43%–100% B; 39–44 min, 100%–0% B; and 44–47 min, 0% B. A total of 20 fractions were collected according to the peak type and time. The samples were dissolved in mass spectrometry loading buffer after concentration by vacuum centrifugation. 10 × iRT peptides were then proportionally added to the samples and mixed for second-dimensional analysis.

The chromatographic separations were conducted using easy nLC-1200 (Thermo, United States). The final solution was injected into a C_18_ column (75 μm × 25 cm, Thermo, United States) at a rate of 300 nl/min. The mobile phases were composed of 0.1% aqueous formic acid (A) and 2% acetonitrile in water (B). The gradient elution program was defined as follows: 0–1 min, 0%–6% B; 1–63 min, 6%–23% B; 63–77 min, 23%–29% B, 77–86 min, 29%–38% B, 86–88 min, 38%–48% B, 88–89 min, 48%–100% B, 89–95 min, 100% B, and 95–96 min, 100%–0% B. Mass spectra were recorded on Q Exactive HF-X mass spectrometer (Thermo, United States) across a range of 350–1300 Da with accurate mass measurements of all mass peaks. The DDA mode was used for data acquisition, and the top-20 precursor ions were selected for secondary fragmentation.

#### 2.9.2 SWATH mass spectrometry detection for individual sample

Equal amounts of desalted peptides were dissolved with mass spectrometry loading buffer. 10 × iRT peptides were then proportionally added to the solution and mixed before SWATH analysis. The same previously described conditions (section 2.9.1 second-dimensional analysis) were applied to both the chromatographic and mass spectrometry analyses. SWATH window parameters were shown in Supplementary Table S1.

### 2.10 Differential proteins identification

Proteome Discoverer TM Software 2.4 was used to search for hierarchical library data and build a digital spectrum library. The obtained spectral library was imported to Spectronaut ™ for product ion peak extraction from SWATH raw data. iRT was used to correct the retention time. Six peptides per protein and three product ions per peptide were selected for quantitative analysis. Fold change >1.5 or <0.65 and a two-tailed *p* < 0.05, using software Cytoscape v3.7.2 to screen out the interaction between genes or proteins, were set as cut-off values to designate significant changes in protein expressions.

### 2.11 Quantitative verification of differential protein based on parallel reaction monitoring analysis

Parallel Reaction Monitoring (PRM) is an ion monitoring technique with advantages such as high-resolution and high-precision mass spectrometry. PRM can selectively detect target proteins and peptides to achieve relative/absolute quantification. The chromatographic analysis conditions were the same as those previously described in Section 2.9.1. The gradient program was as follows: 0–64 min, 5%–23% B; 64–80 min, 23%–29% B; 80–90 min, 29%–38% B; 90–92 min, 38%–48% B; 92–93 min, 48%–100% B; and 93–120 min 100% B. The mass spectrometry analysis was performed using Q Exactive HF-X (Thermo, United States). Mass spectra were recorded across a range of 300–1500 Da. The acquisition mode was PRM mode.

## 3 Results

### 3.1 Histological evaluations

**FIGURE 1:**
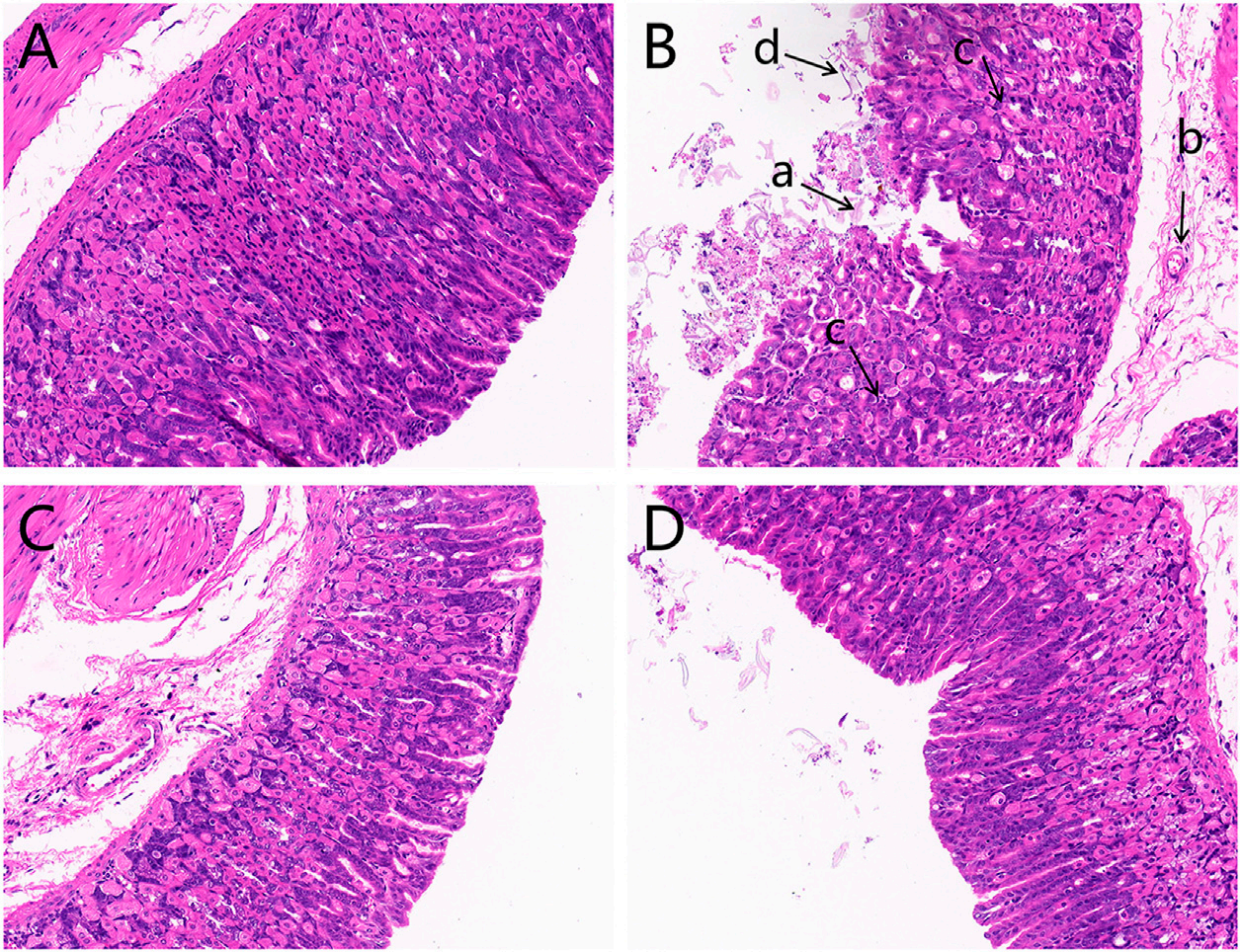
Histological images of hematoxylin and eosin-stained gastric mucosa (magnification ×200). **(A)** Control group; **(B)** Model group; **(C)** OPZ group; **(D)** DF group. (a) Epithelial cell loss; (b) inflammatory cell infiltration; (c) disorganized glandular structures; (d) gastric mucosal hemorrhage.

The histopathological evaluation of gastric mucosal tissues after light microscope observations is displayed in Figure 1. The results indicated that contrary to the control group, which exhibited an integrated gastric mucosa with normal glandular structures, the model group had significant mucosal damages characterized by disorganized glandular structures, a mass of epithelial cell loss, gastric mucosal hemorrhage, and inflammatory cell infiltration. Additionally, OPZ group showed reduced desquamation of epithelial cells compared to the model group. Furthermore, the DF group had far lesser mucosal injuries than both the model and OPZ groups.

### 3.2 Evaluation of the anti-mucosal injury activities of *Dendrobium fimbriatum* through measurements of key cytokine levels

**FIGURE 2:**
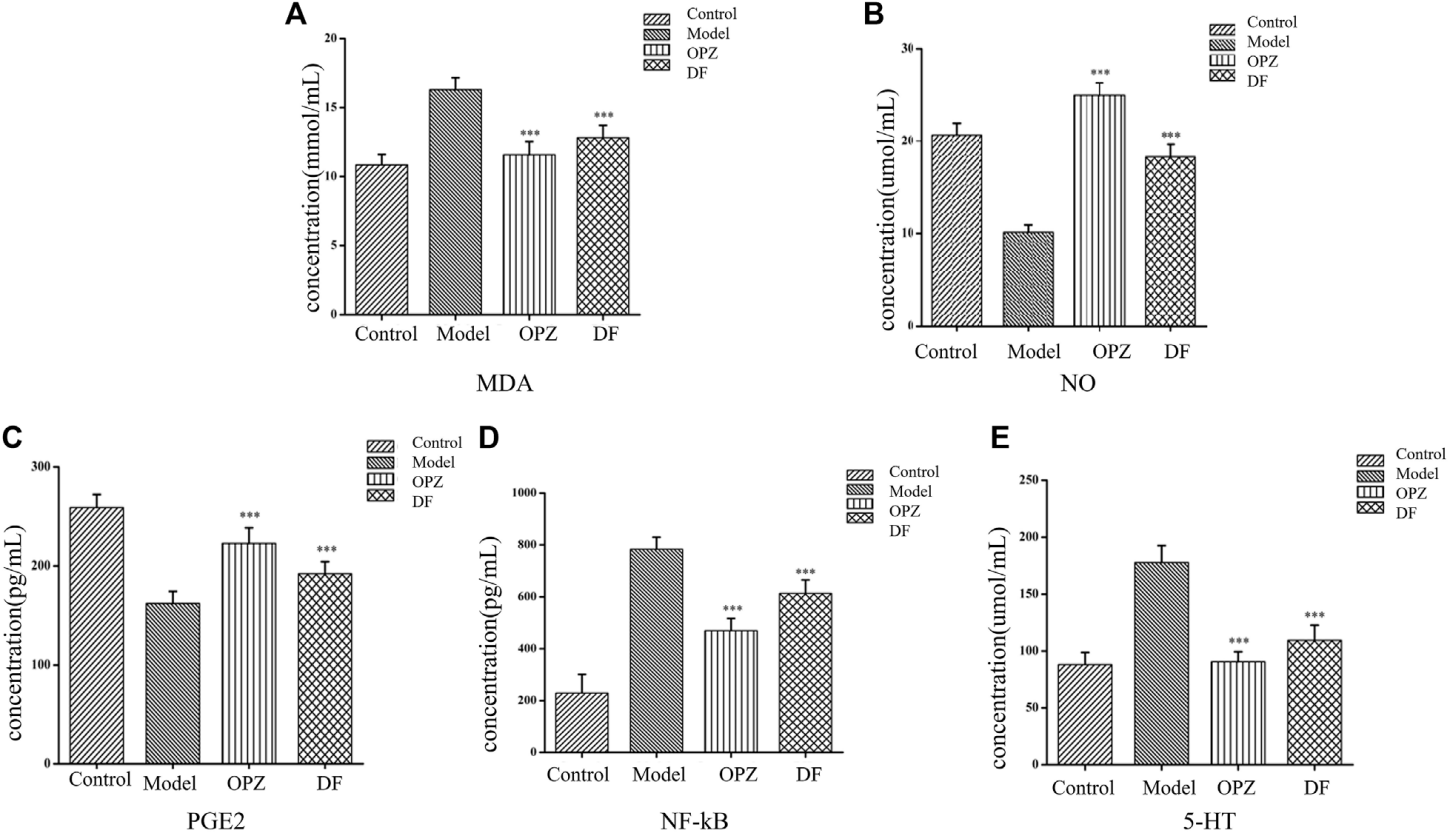
Effect of DF on the contents of MDA **(A)**, NO **(B)**, PGE2 **(C)**, NF-κB **(D)**, as and 5-HT **(E)** in stomach of mice subjected to CTX-induced gastric ulcer. Data was expressed as mean ± SD (*n* = 10). Data with different letters showed significant difference from each other (*p < 0.05, **p < 0.01, ***p < 0.001).

Excessive inflammatory factors are expressed in the gastric tissue of rats with gastric injury induced by CTX. NO, MDA, PGE2, 5-HT, and NF-κB could be served as important indicators in the evaluation of gastric mucosal damage. As shown in Figures 2B,C, subjects in the model group exhibited a significant decrease in NO and PEG2, compared to those in the control group. However, administration of both DF and OPZ can significantly increase tissue NO and PEG2 contents (*p* < 0.001). MDA levels are reflective of the severity of free radical-mediated cell damage. As shown in Figure 2A, compared with the control group, the level of MDA in the model group was significantly increased. In contrast, MDA contents were markedly reduced in the OPZ, DF groups compared to the model group (*p* < 0.001). As presented in Figure 2D, subjects in the model group had significantly increased NF-κB levels compared to those in the control group, a trend alleviated by the administration of DF (*p* < 0.001). Yet, it is worth mentioning that NF-κB expressions in DF remained higher than those of the OPZ group. 5-HT is another potential key factor in gastric mucosal protection. Indeed, 5-HT can induce mucosal damage by inhibiting gastric acid secretion and changing mucosal blood flow (Chauhan and Kang, 2015). Herein, the results indicated that compared with the control group, the model group had significantly increased 5-HT expressions (Figure 2E), while the administration of DF had significantly alleviated 5-HT overexpression in stomach tissues (*p* < 0.001).

### 3.3 Multivariate analysis of the metabolomics data

Stomach tissue samples from the four groups were collected for metabolites detection using UHPLC-Q Exactive-MS. The PCA scores of each group based on the positive and negative ion mode are shown in Supplementary Figure S1, which indicated that the metabolic profiles of the four groups presented a certain clustering trend. The OPLS-DA score chart and S-plot chart between each 2 groups are shown in Supplementary Figure S2. The OPLS-DA score chart showed that plots corresponding to the control and model groups were scattered within two different regions. It definitively confirmed the successfulness of the CTX induced gastric mucosal injury model. The DF group and model group tend to be tightly and independently clustered. This finding demonstrated that there were significant differences in metabolites between the two groups. Additionally, the OPLS-DA scores plots of the OPZ group deviated from those of the model group, indicating that the omeprazole’s anti-gastric mucosal damage caused significant changes in the metabolic profile of the mice.

### 3.4 Differential metabolites identification

Metabolomics can simultaneously monitor multiple metabolic pathways and extrapolate their underlying regulatory processes through the identification of up-regulated or down-regulated marker metabolites. Therefore, VIP >1 and *p* < 0.05 were used to identify differential metabolites using a combination of *t*-test and OPLS-DA analysis. As a result, a total of 74 differential metabolites were identified (Supplementary Figure S1) after the evaluation, such as amino acids, lipids, and most of them were lipids. It is worth mentioning that changes in trend within the model group were compared with those of the control group to uncover mucosal injury-related marker metabolites. Meanwhile, trend changes within the DF group were compared with those of the model group to identify marker metabolites reflective of the protective properties of DF.

### 3.5 Differential proteins identification

**FIGURE 3:**
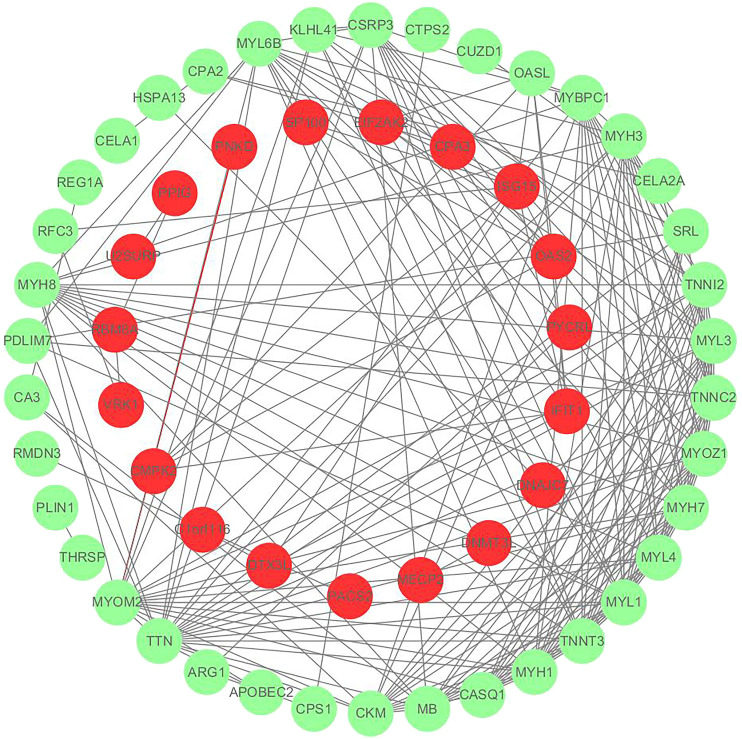
Protein interaction network diagram, red represents down-regulated protein and green represents up-regulated protein.

We utilized the *t*-test function in the Rpackage to evaluate differences between samples. In this project, *p* < 0.05, FC < 0.67, and FC > 1.50 were used to identify differential proteins. A total of 107 differential proteins were identified and are shown in Supplementary Figure S2. Among them, there are 57 differential proteins that interact with each other as shown in Figure 3, and 50 differential proteins are free proteins. Most proteins play an anti-gastric mucosal injury role through interaction. Trend changes within the model group were compared with those of the control group to identify mucosal injury-related target proteins, for example, Ckm, Arg1, Ctps2, etc. Additionally, mucosal protective properties of DF were evaluated through analysis of their target proteins after comparison of trend changes between the DF and the model groups.

### 3.6 Molecular function and pathway analyses

**FIGURE 4:**
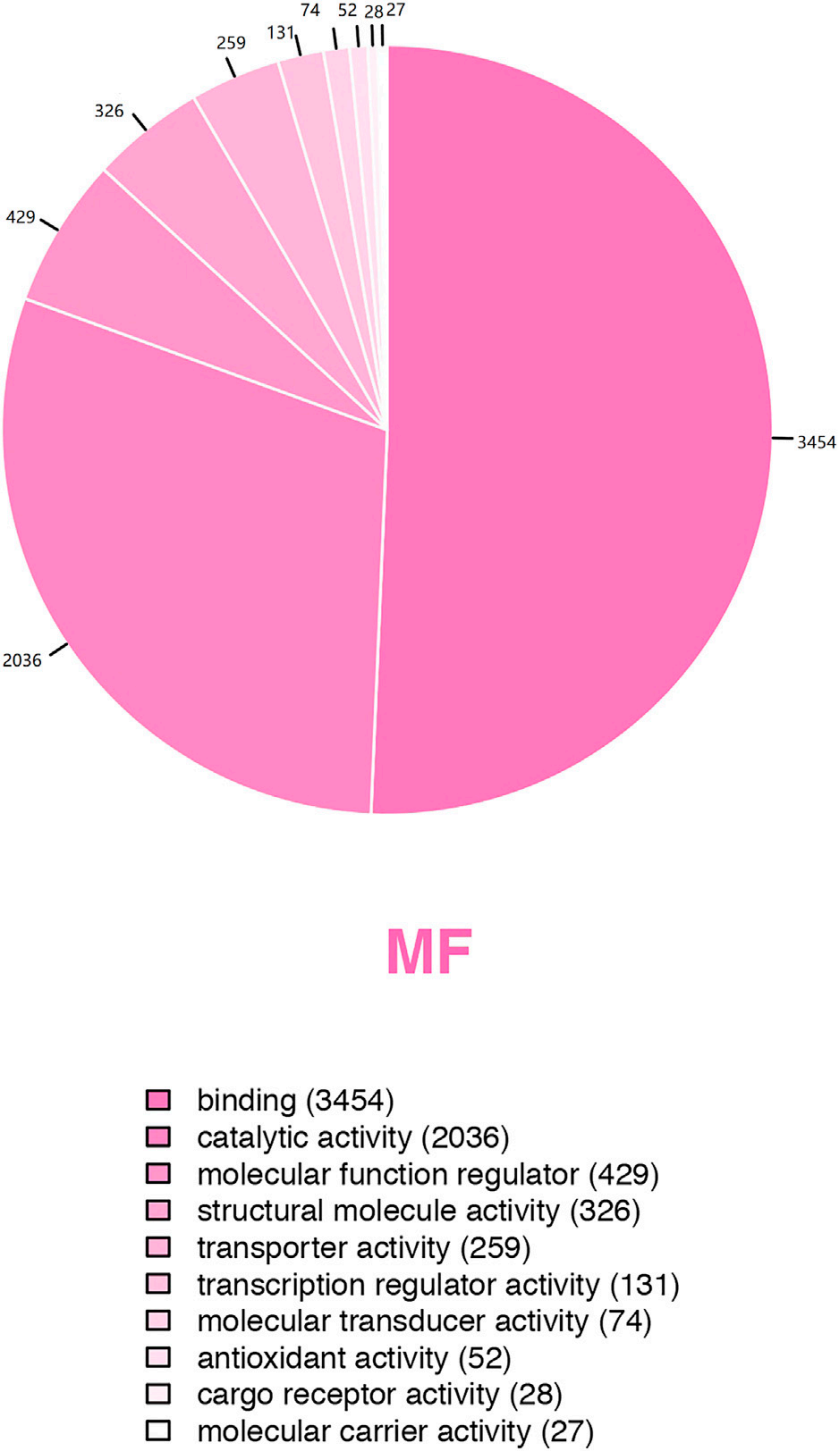
Pie chart of GO secondary classification statistics of all detected proteins.

**FIGURE 5:**
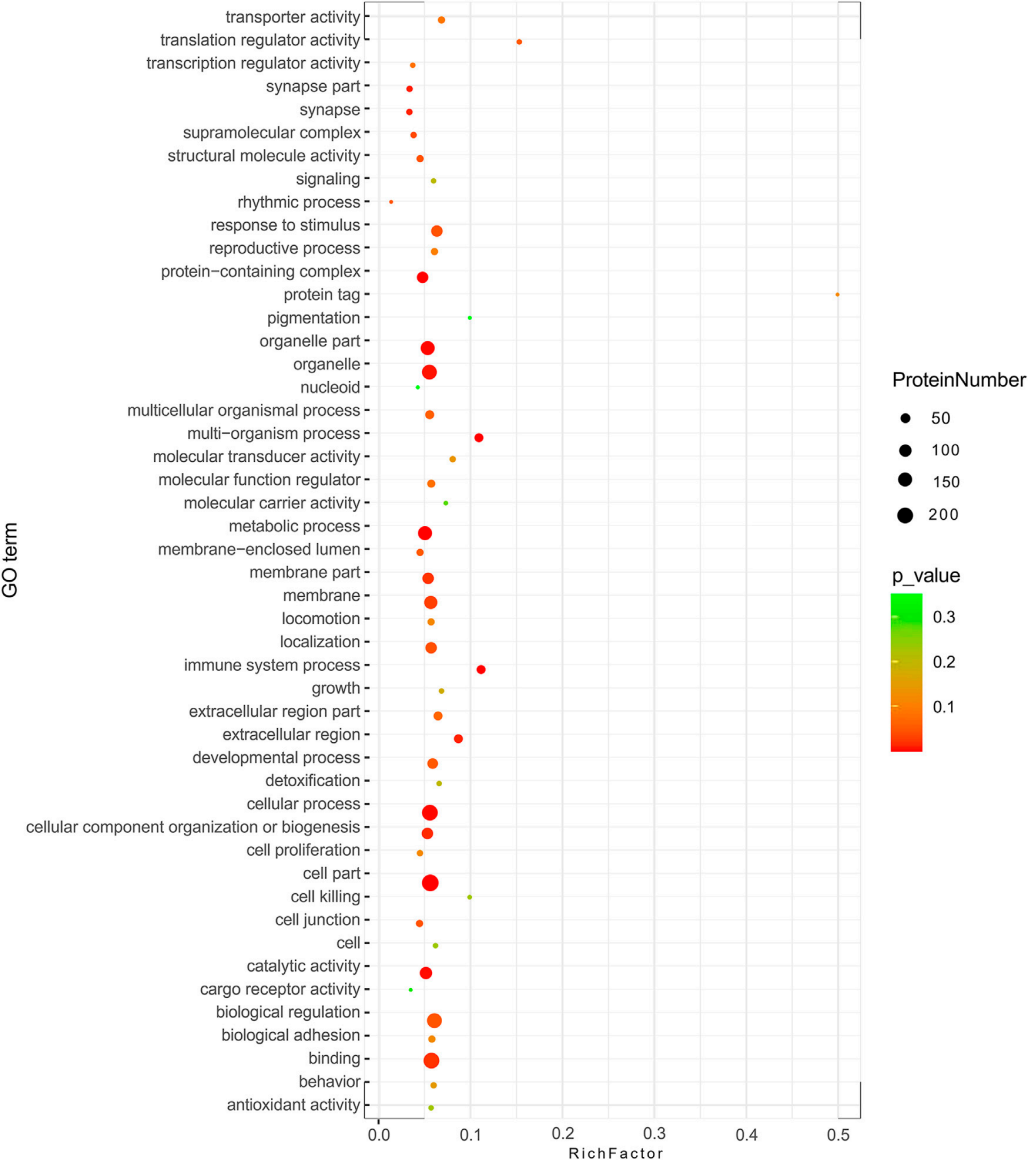
KEGG enrichment bubble chart for the differential proteins-involved pathways.

GO analysis showed the biological functions of all proteins obtained. The proteins were categorized according to their molecular function (MF), protein can be classified into binding, catalytic activity, molecular function regulator, etc. We found that over 50% of the obtained proteins were associated with binding functions (Figure 4). Then enrichment analysis was performed using KEGG software. Each bubble in the KEGG enrichment bubble chart of differential proteins represents a pathway. The abscissa, color, and bubble size respectively indicate the enrichment rate, the significance of enrichment, and the number of enriched proteins within the considered pathway. The more protein is enriched, the larger the bubble **(** Figure 5 **)**. The KEGG pathway enrichment analysis reflected arginine and proline metabolisms, arginine biosynthesis, nitrogen metabolism, DNA replication, and other processes, which may be related to the mechanism of gastric mucosal injury.

### 3.7 Integrative analysis of proteomics and metabolomics

**FIGURE 6:**
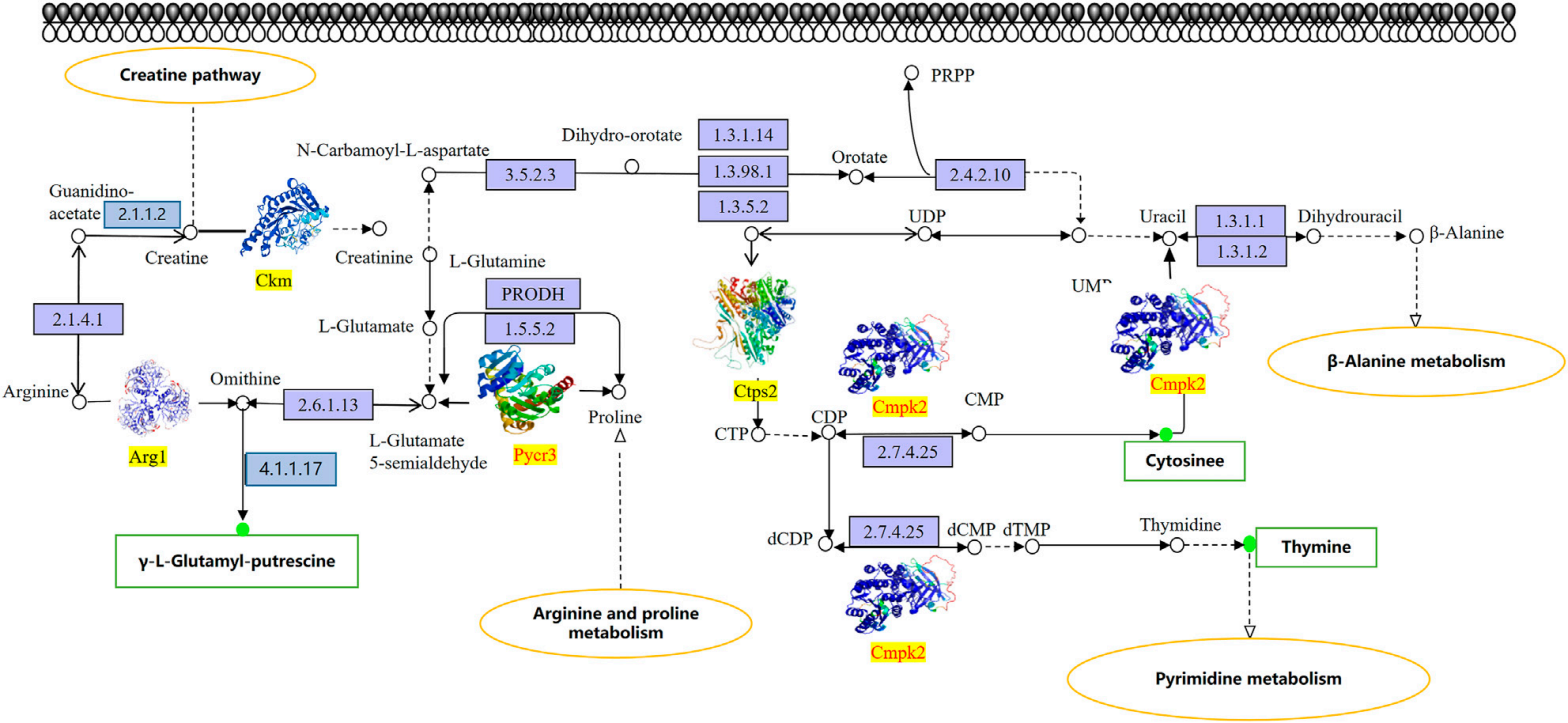
KEGG pathway map of differential metabolites and protein. The box in the figure represents the gene product and the circle represents the metabolite. All gene products in the blue background box reflect background proteins, Ckm, Arg1, and Ctps2 are up-regulated proteins, Pycr3 and Cmpk2 are down-regulated proteins. The metabolites seen in the red/green circles are detected differential metabolites, the red circles indicate up-regulated metabolites, and the green circles indicate down-regulated metabolites.

We proceeded to identify changes in KEGG pathways on mice with chemotherapy drugs to facilitate understanding of the anti-mucosal injury mechanisms of DF. The results showed that a total of three KEGG pathways listed as the creatine pathway, arginine and proline metabolism, and pyrimidine metabolism were significantly altered in the study subjects’ stomach tissue samples (Figure 6). Additionally, three differential proteins (Ckm, Arg1, and Ctps2) and three differential metabolites (γ-L-glutamyl-putrescine, cytosine, and thymine) were down-regulated in the DF group and up-regulated in the model group. In contrast, two differential proteins Pycr3 and Cmpk2 were up-regulated in the DF group and down-regulated in the model group. PRM was then used to quantify the differential proteins involved in the studied pathways (Table 1), which showed that the verification result was completely consistent with the measurement result.

**TABLE 1:** PRM relative quantitative verification results of some differential proteins.

Peptide	Name	Model	FC(Model/Control)	DF	FC (DF/Model)	OPZ	FC (OPZ/Model)
DIVYIGLR	Arg1	↑^#^	2.28	↓**	0.47	Statistically Insignificant	1.44
ANEELAGVVAEVQK	Arg1	↑^#^	1.93	↓*	0.41	Statistically Insignificant	1.37
DLFDPIIQDR	Ckm	↑^###^	1.92	↓*	0.48	Statistically Insignificant	0.57
VLTPDLYNK	Ckm	↑^###^	2.11	↓**	0.55	Statistically Insignificant	0.46
SYALCVPLAPGEGCGPR	Cmpk2	↓^##^	0.60	↑*	1.86	↑**	1.78
IAAQTLLGTAK	Pycr3	↓^#^	0.44	↑**	1.98	↑**	1.93
GLGLSPDLIVCR	Ctps2	↑^###^	1.79	↓**	0.47	↓*	0.37

^#^ *p* < 0.05, ^##^ *p* < 0.01, ^###^ *p* < 0.001 compared with control; *p < 0.05, **p < 0.01, ***p < 0.001 compared with model; “↑”, increase in signal; “↓”, decrease in signal; Data are expressed as mean ± SD (*n* = 5).

## 4 Discussion

Studies on homologous plants of medicines and foods are increasing in recent years. As a medicinal and edible plant, DF possesses good gastric mucosal protective properties. The present study aimed to evaluate the gastroprotective effects of DF on CTX-induced gastric mucosal injury. Previous studies have demonstrated that gastric mucosal injury was characterized by disorganized structure of the glandular, a mass of epithelial cell loss, gastric mucosal hemorrhage and inflammatory cell infiltration (Chauhan and Kang, 2015; Qin et al., 2017; Zhang et al., 2018). These results were in consistence with our findings in current study. Sever pathological injury occurred in a mice model of gastric injury induced by CTX in our study, and pretreatment with DF could obviously alleviate gastric mucosal injury status. Cytokines play crucial roles towards regulating and mediating inflammatory and immune responses. Existing evidence has proved that the levels of NF-κB, MDA, 5-HT, PGE2, and NO were involved in the healing the gastric mucosal damage (Ko et al., 1998; Beserra et al., 2011; Li et al., 2011; Arab et al., 2015; Chang et al., 2015). In current study, DF treatment could significantly reduce the concentrations of NF-κB, MDA, and 5-HT in the stomach tissue of mice, nonetheless, dramatically increase the contents of PGE2 and NO, agreeing well with previous studies. Therefore, we speculate that the potential gastroprotective effect of DF might rely on regulating the levels of NF-κB, MDA, 5-HT, PGE2, and NO in the serum. The above studies have disclosed the anti-gastric mucosal damage effect of the DF, but its mechanism needs to be further explored.

The association and integration of differential metabolites and proteins were analyzed by targeting analysis and SWATH approach to construct an overall protein-metabolite regulatory network, from which three differential metabolites (γ-*L*-glutamyl-putrescine, cytosine, and thymine) and three proteins (Ckm, Arg1, and Ctps2) were found to be up-regulated and two down-regulated proteins (Pycr3 and Cmpk2). Ckm is the M-type of the creatine kinase (CK) isoform that reversibly catalyzes the process of phosphate transfer between ATP and individual reactions. As being seen in the creatine pathway, creatinine is synthesized by a two-step process. Firstly, glycine amidino transferase (GATM, EC 2.1.4.1) is reacted in the kidney or pancreas to obtain guanidinoacetate, followed by converting to creatine under the catalysis of *S-*adenosyl-L-metheonine*-N-*guanidinoacetate methyl transferase (EC 2.1.1.2). Creatine kinase (CK) has four different isoforms, the pathway involved in this study was M-type (Ckm). In the study, the level of Ckm in the model group was significantly increased, whilst the trend of DF group was opposite, inhibiting the occurrence of the creatine pathway. Studies have shown that the level of CK in stomach tissue is related to inflammation. The CK-induced response mediated by HIF may be directly related to the antioxidant pathway of hypoxic cells, and the abnormal regulation of biological energy caused by impaired CK shuttle may increase the barrier permeability of inflammatory mucosa (Glover et al., 2013). Noteworthily, significant increment in B-type creatinase activity was detected in the serum of patients with gastric cancer disease and the changes in creatine kinase isoenzyme activity were related to various neoplastic states (Hirata et al., 1989). In addition, there is a great risk of transforming into gastric cancer when the degree of gastric mucosal damage is severe, resulting in that the gastric mucosal damage is inextricably linked with the occurrence of gastric cancer. These results were consistent with the results obtained in current study, suggesting that Ckm may become a target for gastrointestinal diseases and may be related to cell energy metabolism and inflammation.

Arg1 is the key element of the urea cycle when converting *L*-arginine to urea and L-ornithine. On the one hand, ODC (EC 4.1.1.17) catalyzes the conversion of ornithine to putrescine, then PuuA uses putrescine as a nitrogen source and carbon source to decompose putrescine, and it is also the enzyme that catalyzes the last step in proline biosynthesis. Previous studies have shown that putrescine is closely related to gastric mucosal epithelial cell migration, proliferation, differentiation, regeneration, and tumor deterioration (Kitao et al., 2010). In current study, the level of Arg1 and the downstream product of putrescine and γ-L-glutamyl-putrescine, were significantly increased in the model group; however, the opposite trend occurred in DF group, which was consistent with the results of the previous study (Manning et al., 2017). On the other hand, *L*-ornithine was further metabolized to proline by Pycr3. In this study, we found that Pycr3 showed a down-regulation trend in the model group and an up-regulation trend in the DF group, nonetheless. Pycr3 is an enzyme being responsible for catalyzing the last step in the biosynthesis of proline. Proline plays an important role in protein synthesis, metabolism, nutritional supplement, wound healing, antioxidant response, and immune response (Wu et al., 2011). Therefore, we speculate that the up-regulation of Pycr3 may play a role in resisting gastric mucosal damage by affecting the synthesis of proline.

In the model group of this study, a down-regulated protein (Cmpk2) and an up-regulated protein (Ctps2) were involved in pyrimidine metabolism, ultimately leading to that the levels of cytosine and thymine were up-regulated; however, opposite trends occurred in the DF group. Interestingly, it is similar to the anticancer principle of fluorouracil (5-FU). 5-FU is the most widely used anti-tumor drug in clinical applications. Many studies have revealed that 5-FU can be used for the treatment of pancreatitis (Johnson et al., 1973). 5-FU can reduce anti-inflammatory cytokines in pancreatitis, such as IL-10 and TGF-β. This led us to speculate that the anti-gastric mucosal damage mechanism of DF was similar to 5-FU against pancreatitis. In summary, Ckm, Arg1, Ctps2, Pycr3, and Cmpk2 may be the target proteins of DF against gastric mucosal injury, and γ-L-glutamyl-putrescine, cytosine and thymine may be the determinant metabolites of DF against gastric mucosal injury. The accuracy of the results was verified by PRM analysis.

The combination between proteomics and metabolomics presented in this paper which can focus on research directions and screen potential targets and biomarkers provide a more accurate, more credible research direction. These results provide new insights into the pathology of gastroprotective activity of DF, it is also helpful for the further development of functional food discovery, clinical diagnosis and treatment of gastric mucosal injury, and contributes to the more reasonable development and utilization of DF*.*

## 5 Conclusion

In conclusion, pathological analysis in this study showed that DF has an anti-gastric mucosal damage effect, and the combination of proteomics and metabolomics were employed to clarify in depth the mechanisms. The changes of proteins and metabolites in the gastric tissues of the control, model, OPZ and DF groups were detected, and 74 differential metabolites together with 107 differentially expressed proteins were captured and tentatively identified. The anti-gastric mucosal injury mechanism of DF identified three metabolic pathways through three upregulated differential metabolites (γ-L-glutamyl putrescine, cytosine, and thymine), three upregulated proteins (Ckm, Arg1, and Ctps2), and two downregulated proteins (Pycr3 and Cmpk2) that contribute to the fight against gastric mucosal injury. The metabolism and protein deviations can be restored after DF treatment. These results suggested that DF plays a pivotal role against gastric mucosal injury treatment through the downregulation and upregulation of endogenous metabolite and protein levels. Our study provides an important scientific basis and theoretical guidance for further research on the development of DF functional foods, which is beneficial to the further development of *Dendrobium* industry.

## Data Availability

The datasets presented in this study can be found in online repositories. The names of the repository/repositories and accession number(s) can be found in the article/Supplementary Material.
