# Supplementary material for: Combined metabolomics and proteomics to reveal beneficial mechanisms of Dendrobium fimbriatum against gastric mucosal injury
Source: Front Pharmacol. 2022 Aug 30;13:948987. doi: 10.3389/fphar.2022.948987 (PMC9468276; doi:10.3389/fphar.2022.948987)
Supplement: Supplementary file 2 [file DataSheet1.doc]

Supplementary Files

**Combined metabolomics and proteomics to reveal beneficial mechanisms of *Dendrobium fimbriatum* against gastric mucosal injury**

**Jing Sun^1^****^†^, Peng**-**fei Liu^1,2†^, Jia**-**ni Liu^1^, Cong Lu^1^, Li**-**tao Tong^1^, Yong**-**quan Wang^1^, Jia**-**meng Liu^1^, Bei Fan^1*^ and Feng**-**zhong Wang^1*^**

^1^ Risk Assessment Laboratory of Agricultural Products Processing Quality and Safety, Key Laboratory of Agricultural Products Quality and Safety Collection, Storage and Transportation Control (Ministry of Agriculture and Rural Affairs), Institute of Agricultural Products Processing, Chinese Academy of Agricultural Sciences, Beijing, China

^2^ School of Pharmacy, Hunan University of Chinese Medicine, Changsha, China

*** Correspondence:**Corresponding Author
Tel/Fax: 86-10-62817417, E-mail: fanbei517@163.com (Bei Fan)

Tel/Fax: 86-10-62815977, E-mail: [wangfengzhong@sina.com](mailto:wangfengzhong@caas.cn) (Feng-zhong Wang)

† These authors have contributed equally to this work and share first authorship

| **No.** | **Content** |
| --- | --- |
| **Figure S1** | PCA scores plots of stomach tissue samples obtained from 5 groups based on UHPLC-Q Exactive-MS data |
| **Figure S2** | OPLS-DA scores plots and S-Plot of stomach tissue samples based on UHPLC-Q Exactive-MS data. The red markers were considered as potential biomarkers. (A) Control group and model group; (B) DF group and model group; (C) OPZ group and model group |
| **Table S1** | Differential metabolites detected *via* UHPLC -Q Exactive among the control, CTX model injury, Dendrobium fimbriatum extract and positive groups |
| **Table S2** | Detected differential proteins via SWATH analysis |


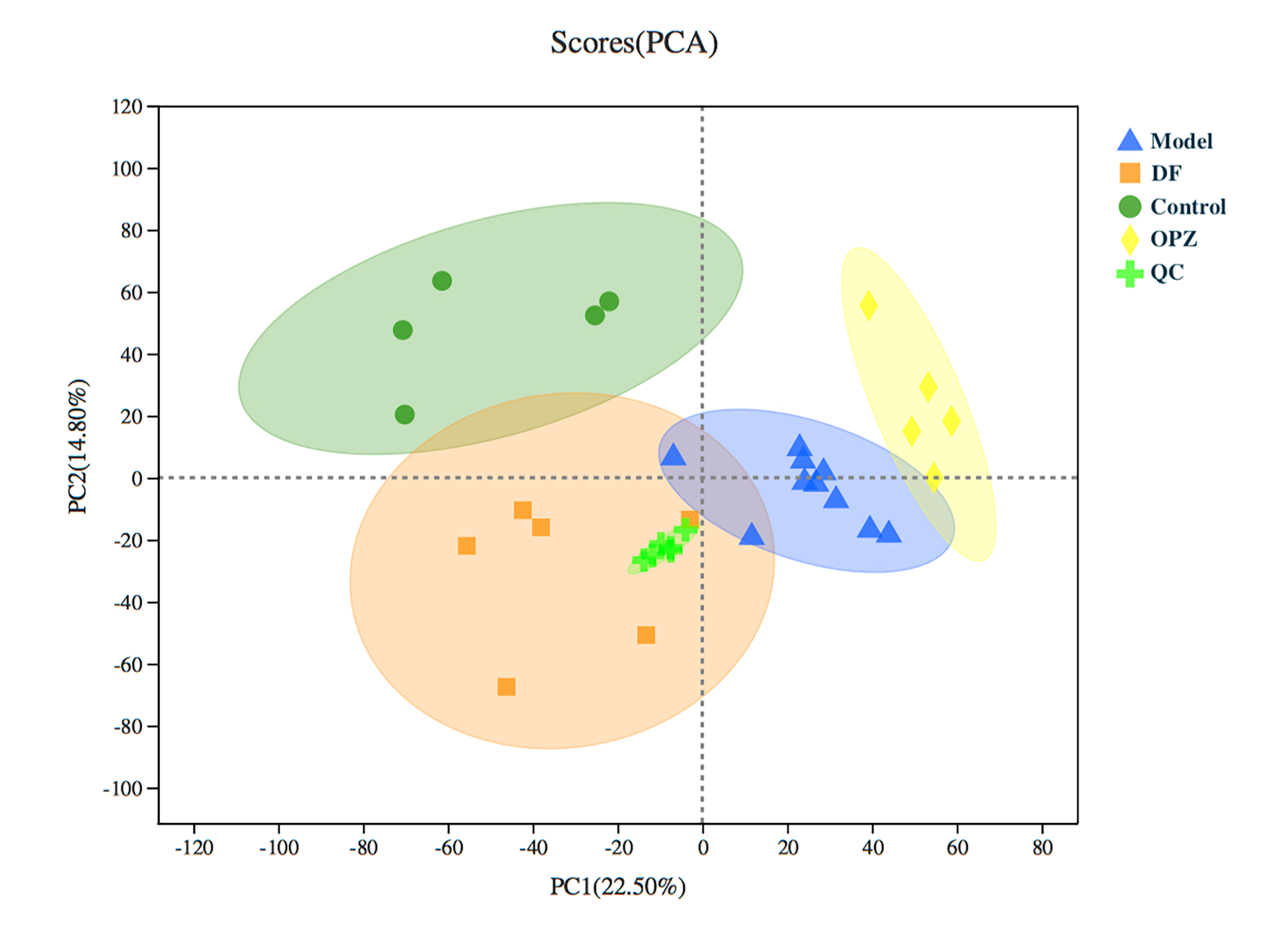


**Figure S1.** PCA scores plots of stomach tissue samples obtained from 5 groups based on UHPLC-Q Exactive-MS data


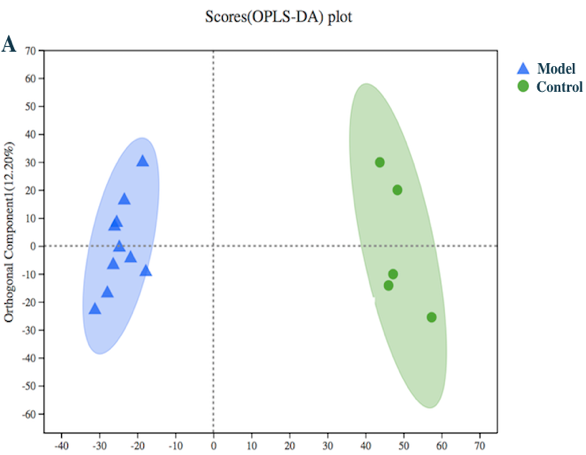

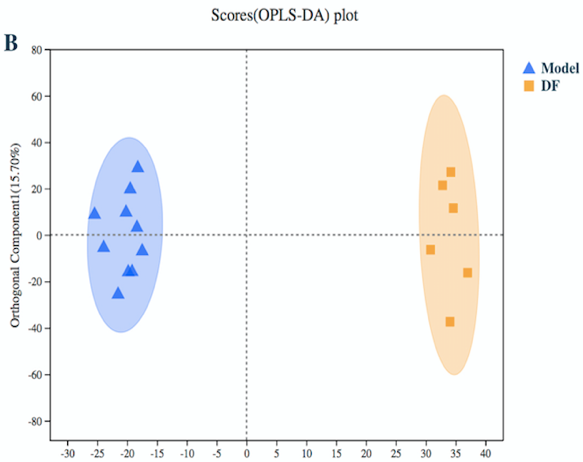

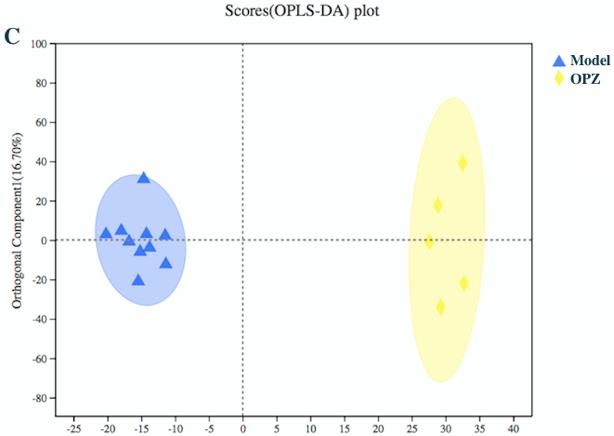


**Figure S2.** OPLS-DA scores plots and S-Plot of stomach tissue samples based on UHPLC-Q Exactive-MS data. The red markers were considered as potential biomarkers. (A) Control group and model group; (B) DF group and model group; (C) OPZ group and model group

**Table S1**

Differential metabolites detected via UHPLC -Q Exactive among the control, CTX model injury, *Dendrobium fimbriatum* extract and positive groups

| NO | Mode | Metabolite | M/Z | Formula | RT | Model | VIP | DH | VIP |
| --- | --- | --- | --- | --- | --- | --- | --- | --- | --- |
| 1 | pos | Sucrose | 365.10 | C12H22O11 | 0.60 | ↓^###^ | 2.66 | ↑** | 2.07 |
| 2 | pos | Methionyl-Threonine | 292.13 | C9H18N2O4S | 1.75 | ↑^##^ | 1.10 | ↓** | 1.34 |
| 3 | pos | (2S)-2-{[1-(R)-Carboxyethyl]amino}pentanoate | 190.11 | C8H15NO4 | 1.93 | ↓^##^ | 1.21 | ↑* | 1.22 |
| 4 | pos | 3-hydroxy-6-methyl-2H-chromen-2-one | 177.05 | C10H8O3 | 2.48 | ↓^##^ | 1.11 | ↑*** | 1.56 |
| 5 | pos | Antibiotic SB 202742 | 353.25 | C24H34O3 | 5.15 | ↑^##^ | 1.59 | ↓*** | 2.60 |
| 6 | pos | 3a,7a,12b-Trihydroxy-5b-cholanoic acid | 373.27 | C24H40O5 | 5.95 | ↑^##^ | 1.29 | ↓*** | 2.33 |
| 7 | pos | Ricinoleic acid | 299.26 | C18H34O3 | 6.25 | ↑^###^ | 2.19 | ↓* | 1.81 |
| 8 | pos | MG(0:0/16:0/0:0) | 313.27 | C19H38O4 | 6.93 | ↑^#^ | 1.98 | ↓* | 2.23 |
| 9 | pos | 3beta-3-Hydroxy-18-lupen-21-one | 441.37 | C30H48O2 | 7.40 | ↑^###^ | 2.08 | ↓* | 1.20 |
| 10 | pos | Oleoyl glycine | 381.31 | C20H37NO3 | 8.85 | ↑^###^ | 3.89 | ↓* | 1.57 |
| 11 | pos | LysoPE(18:0/0:0) | 963.64 | C23H48NO7P | 8.89 | ↓^###^ | 1.43 | ↑** | 1.50 |
| 12 | pos | 7-Dehydrodesmosterol | 383.33 | C27H42O | 8.89 | ↑^###^ | 2.01 | ↓* | 1.53 |
| 13 | pos | PE(18:0/0:0) | 482.32 | C23H48NO7P | 8.91 | ↓^###^ | 1.03 | ↑* | 1.04 |
| 14 | pos | 1-Stearoylglycerophosphoserine | 570.28 | C24H48NO9P | 8.97 | ↓^###^ | 1.43 | ↑** | 1.04 |
| 15 | pos | 1-oxoprevitamin D3 | 399.32 | C27H42O2 | 9.01 | ↑^###^ | 2.35 | ↓* | 1.39 |
| 16 | pos | (3beta,22R,23R,24S)-3,22,23-Trihydroxystigmastan-6-one | 463.38 | C29H50O4 | 9.45 | ↑^###^ | 1.93 | ↓** | 1.70 |
| 17 | pos | PC(16:1(9Z)/22:6(4Z,7Z,10Z,13Z,16Z,19Z)) | 826.53 | C46H78NO8P | 9.91 | ↓^###^ | 3.01 | ↑*** | 2.26 |
| 18 | pos | PC(18:2(9Z,12Z)/22:6(4Z,7Z,10Z,13Z,16Z,19Z)) | 830.56 | C48H80NO8P | 9.93 | ↓^###^ | 2.55 | ↑** | 1.72 |
| 19 | pos | PC(14:0/22:5(7Z,10Z,13Z,16Z,19Z)) | 802.53 | C44H78NO8P | 10.02 | ↓^###^ | 2.64 | ↑** | 1.67 |
| 20 | pos | PC(14:0/20:4(8Z,11Z,14Z,17Z)) | 754.54 | C42H76NO8P | 10.02 | ↓^###^ | 2.27 | ↑** | 1.66 |
| 21 | pos | PC(14:1(9Z)/18:1(9Z)) | 730.54 | C40H76NO8P | 10.11 | ↓^###^ | 2.16 | ↑** | 1.59 |
| 22 | pos | PC(16:0/18:3(9Z,12Z,15Z)) | 756.55 | C42H78NO8P | 10.19 | ↓^###^ | 2.53 | ↑** | 1.69 |
| 23 | pos | PC(22:6(4Z,7Z,10Z,13Z,16Z,19Z)/P-16:0) | 790.57 | C46H80NO7P | 10.26 | ↓^###^ | 1.51 | ↑** | 1.40 |
| 24 | pos | PC(16:0/20:4(5Z,8Z,11Z,14Z)) | 782.57 | C44H80NO8P | 10.28 | ↓^###^ | 2.09 | ↑* | 1.56 |
| 25 | pos | PC(16:0/18:2(9Z,12Z)) | 758.57 | C42H80NO8P | 10.37 | ↓^###^ | 2.44 | ↑** | 1.98 |
| 26 | pos | PE(P-18:1(11Z)/20:5(5Z,8Z,11Z,14Z,17Z)) | 748.53 | C43H74NO7P | 10.39 | ↓^###^ | 3.17 | ↑*** | 3.22 |
| 27 | pos | PE(14:1(9Z)/18:1(9Z)) | 720.51 | C37H70NO8P | 10.41 | ↑^###^ | 1.56 | ↓*** | 1.26 |
| 28 | pos | PC(18:0/22:6(4Z,7Z,10Z,13Z,16Z,19Z)) | 856.58 | C48H84NO8P | 10.43 | ↓^###^ | 2.27 | ↑** | 1.91 |
| 29 | pos | Phosphocholine | 184.07 | C5H14NO4P | 10.52 | ↓^###^ | 1.39 | ↑** | 1.60 |
| 30 | pos | PE(P-18:1(11Z)/18:3(6Z,9Z,12Z)) | 724.53 | C41H74NO7P | 10.56 | ↓^###^ | 2.83 | ↑*** | 2.66 |
| 31 | pos | PS(16:0/24:1(15Z)) | 810.60 | C46H88NO10P | 10.58 | ↓^###^ | 2.54 | ↑** | 2.14 |
| 32 | pos | PC(16:0/18:1(11Z)) | 760.58 | C42H82NO8P | 10.69 | ↓^###^ | 2.50 | ↑*** | 2.41 |
| 33 | pos | PC(18:0/22:4(7Z,10Z,13Z,16Z)) | 838.63 | C48H88NO8P | 10.82 | ↓^###^ | 3.51 | ↑*** | 3.49 |
| 34 | pos | PC(O-16:0/18:2(9Z,12Z)) | 744.59 | C42H82NO7P | 10.89 | ↓^###^ | 2.31 | ↑** | 2.01 |
| 35 | pos | PE(P-18:1(11Z)/22:6(4Z,7Z,10Z,13Z,16Z,19Z)) | 774.54 | C45H76NO7P | 10.95 | ↓^###^ | 3.94 | ↑*** | 4.34 |
| 36 | pos | PC(o-18:1(11Z)/16:0) | 746.60 | C42H84NO7P | 10.97 | ↓^###^ | 1.91 | ↑** | 1.87 |
| 37 | pos | PE(P-18:1(11Z)/16:0) | 702.54 | C39H76NO7P | 11.11 | ↓^###^ | 2.44 | ↑*** | 2.51 |
| 38 | neg | 3-Oxoglutaric acid | 191.02 | C5H6O5 | 0.63 | ↓^###^ | 1.17 | ↑** | 1.33 |
| 39 | neg | Serylmethionine | 235.08 | C8H16N2O4S | 0.91 | ↑^##^ | 1.34 | ↓** | 1.35 |
| 40 | neg | 3-Methylglutaric acid | 145.05 | C6H10O4 | 1.00 | ↓^##^ | 1.22 | ↑*** | 2.34 |
| 41 | neg | Asparaginyl-Isoleucine | 244.13 | C10H19N3O4 | 1.41 | ↑^#^ | 1.08 | ↓** | 1.22 |
| 42 | neg | Tryptophyl-Asparagine | 317.13 | C15H18N4O4 | 1.77 | ↑^###^ | 1.23 | ↓** | 1.27 |
| 43 | neg | 24,25,26,27-Tetranor-23-oxo-hydroxyvitamin D3 | 403.25 | C23H34O3 | 4.79 | ↑^###^ | 2.51 | ↓* | 2.15 |
| 44 | neg | 15-deoxy-delta-12,14-PGJ2 | 315.20 | C20H28O3 | 4.97 | ↓^###^ | 1.10 | ↑*** | 1.31 |
| 45 | neg | 11beta,20-Dihydroxy-3-oxopregn-4-en-21-oic acid | 419.21 | C22H30O5 | 4.97 | ↓^###^ | 1.05 | ↑*** | 1.19 |
| 46 | neg | 8-iso Prostaglandin A2 | 333.21 | C20H30O4 | 4.97 | ↓^##^ | 1.01 | ↑*** | 1.33 |
| 47 | neg | Alpha-Phenylcyclohexylglycolic acid | 233.12 | C14H18O3 | 5.04 | ↓^##^ | 1.01 | ↑*** | 1.36 |
| 48 | neg | 13'-Hydroxy-gamma-tocotrienol | 471.31 | C28H42O3 | 5.11 | ↑^#^ | 1.23 | ↓*** | 2.12 |
| 49 | neg | 1b,3a,7b-Trihydroxy-5b-cholanoic acid | 405.26 | C24H40O6 | 5.13 | ↑^##^ | 1.38 | ↓*** | 2.47 |
| 50 | neg | 2-benzyl-6-hydroxyheptanal | 485.29 | C14H20O2 | 5.59 | ↑^#^ | 1.17 | ↓* | 1.45 |
| 51 | neg | Glabric acid | 485.33 | C30H46O5 | 5.61 | ↑^##^ | 1.71 | ↓* | 1.50 |
| 52 | neg | (3beta,5alpha,6alpha,7alpha,22E,24R)-5,6-Epoxyergosta-8,14,22-triene-3,7-diol | 471.31 | C28H42O3 | 5.68 | ↑^#^ | 1.12 | ↓* | 1.37 |
| 53 | neg | Cholic acid | 407.28 | C24H40O5 | 5.93 | ↑^#^ | 1.13 | ↓*** | 2.19 |
| 54 | neg | 19alpha-19-Hydroxy-3,11-dioxo-12-ursen-28-oic acid | 469.30 | C29H42O5 | 6.02 | ↑^##^ | 1.41 | ↓** | 1.65 |
| 55 | neg | Coprocholic acid | 471.31 | C27H46O5 | 6.05 | ↑^##^ | 1.53 | ↓* | 1.43 |
| 56 | neg | 3b,12a-Dihydroxy-5a-cholanoic acid | 437.29 | C24H40O4 | 6.09 | ↑^###^ | 1.94 | ↓* | 1.33 |
| 57 | neg | (+)-15,16-Dihydroxyoctadecanoic acid | 315.25 | C18H36O4 | 6.23 | ↑^##^ | 1.96 | ↓* | 1.61 |
| 58 | neg | Gamma-Tocotrienol | 455.32 | C28H42O2 | 6.46 | ↑^###^ | 1.91 | ↓*** | 1.87 |
| 59 | neg | N-[[3-Hydroxy-2-(2-pentenyl)cyclopentyl]acetyl]isoleucine | 306.21 | C18H31NO4 | 6.84 | ↑^##^ | 1.15 | ↑** | 1.49 |
| 60 | neg | (25R)-3beta,4beta-dihydroxycholest-5-en-26-oate(1-) | 453.30 | C27H44O4 | 6.94 | ↑^##^ | 1.46 | ↓** | 1.73 |
| 61 | neg | Buprenorphine glucuronide | 624.31 | C35H49NO10 | 7.59 | ↑^###^ | 1.67 | ↓* | 1.28 |
| 62 | neg | Cytochalasin Ppho | 510.28 | C30H41NO6 | 8.57 | ↓^##^ | 1.20 | ↑** | 1.31 |
| 63 | neg | PC(20:4(5Z,8Z,11Z,14Z)/22:6(4Z,7Z,10Z,13Z,16Z,19Z)) | 898.56 | C50H80NO8P | 9.83 | ↓^###^ | 2.60 | ↑** | 1.63 |
| 64 | neg | DG(15:0/18:1(11Z)/0:0) | 579.50 | C36H68O5 | 9.90 | ↑^##^ | 2.66 | ↓* | 2.01 |
| 65 | neg | PE-NMe(20:2(11Z,14Z)/22:6(4Z,7Z,10Z,13Z,16Z,19Z)) | 874.56 | C48H80NO8P | 9.92 | ↓^###^ | 2.55 | ↑** | 1.62 |
| 66 | neg | PE-NMe(16:0/20:3(5Z,8Z,11Z)) | 800.55 | C42H78NO8P | 10.08 | ↓^###^ | 2.48 | ↑* | 1.38 |
| 67 | neg | PE-NMe(16:0/18:2(9Z,12Z)) | 774.53 | C40H76NO8P | 10.08 | ↓^###^ | 2.13 | ↑* | 1.35 |
| 68 | neg | PE-NMe(18:0/22:6(4Z,7Z,10Z,13Z,16Z,19Z)) | 850.56 | C46H80NO8P | 10.15 | ↓^###^ | 2.58 | ↑** | 1.83 |
| 69 | neg | PE-NMe2(16:0/18:2(9Z,12Z)) | 788.54 | C41H78NO8P | 10.22 | ↓^###^ | 2.00 | ↑** | 1.58 |
| 70 | neg | PE-NMe(18:2(9Z,12Z)/18:0) | 802.56 | C42H80NO8P | 10.35 | ↓^###^ | 2.52 | ↑** | 2.00 |
| 71 | neg | PC(18:2(9Z,12Z)/P-16:0) | 786.57 | C42H80NO7P | 10.51 | ↓^###^ | 2.26 | ↑* | 1.44 |
| 72 | neg | PE-NMe(18:0/22:4(7Z,10Z,13Z,16Z)) | 854.59 | C46H84NO8P | 10.55 | ↓^###^ | 2.54 | ↑** | 2.10 |
| 73 | neg | PE(15:0/22:1(13Z)) | 804.58 | C42H82NO8P | 10.66 | ↓^###^ | 2.32 | ↑** | 1.96 |
| 74 | neg | PE-NMe(18:0/20:2(11Z,14Z)) | 830.59 | C44H84NO8P | 10.69 | ↓^###^ | 2.78 | ↑** | 2.35 |

^#^p<0.05, ^##^p<0.01, ^###^p<0.001 compared with control; ^*^p<0.05， ^**^p<0.01，^***^p<0.001 compared with model; “↑”, increase in signal; “↓”, decrease in signal; Data are expressed as mean ±SD (n=10).

**Table S2**

Detected differential proteins *via* SWATH analysis

| Accession | Gene name | Protein name | | Model | FC（Model /Control） | DF | FC（DF/Model） |
| --- | --- | --- | --- | --- | --- | --- | --- |
| Q9Z2F2 | Oasl2 | 2'-5'-oligoadenylate synthase-like protein 2 | | ↓^#^ | 0.37 | ↑^**^ | 3.46 |
| Q9Z2D6 | Mecp2 | Methyl-CpG-binding protein 2 | | ↓^#^ | 0.38 | ↑^**^ | 2.32 |
| Q9Z2B9 | Rps6ka4 | Ribosomal protein S6 kinase alpha-4 | | ↓^#^ | 0.50 | ↑^**^ | 1.95 |
| Q9WV35 | Apobec2 | C->U-editing enzyme APOBEC-2 | | ↑^###^ | 2.53 | ↓^***^ | 0.50 |
| Q9R0T7 | Try4 | Pancreatic trypsin | | ↑^###^ | 1.59 | ↓^***^ | 0.35 |
| Q9R0Q1 | Sytl4 | Synaptotagmin-like protein 4 | | ↓^###^ | 0.39 | ↑^*^ | 1.53 |
| Q9QYI3 | Dnajc7 | DnaJ homolog subfamily C member 7 | | ↓^#^ | 0.54 | ↑^*^ | 1.76 |
| Q9JK37 | Myoz1 | Myozenin-1 | | ↑^###^ | 8.09 | ↓^***^ | 0.22 |
| Q9DBK7 | Uba7 | E1 ubiquitin-activating enzyme | | ↓^##^ | 0.55 | ↑^*^ | 2.05 |
| Q99P30 | Nudt7 | Peroxisomal coenzyme A diphosphatase NUDT7 | | ↑^#^ | 1.65 | ↓^**^ | 0.58 |
| Q925B0 | Pawr | PRKC apoptosis WT1 regulator protein | | ↓^###^ | 0.42 | ↑^***^ | 2.48 |
| Q91Z83 | Myh7 | Myosin-7 | | ↑^###^ | 3.40 | ↓^***^ | 0.43 |
| Q91XB0 | Trex1 | Three-prime repair exonuclease 1 | | ↓^#^ | 0.53 | ↑^***^ | 1.92 |
| Q91X79 | Cela1 | Chymotrypsin-like elastase family member 1 | | ↑^###^ | 1.68 | ↓^***^ | 0.45 |
| Q8R429 | Atp2a1 | Sarcoplasmic/endoplasmic reticulum calcium ATPase 1 | | ↑^###^ | 2.04 | ↓^***^ | 0.61 |
| Q8R323 | Rfc3 | Replication factor C subunit 3 | | ↑^#^ | 4.02 | ↓^*^ | 0.27 |
| Q8CI43 | Myl6b | Myosin light chain 6B | | ↑^###^ | 5.35 | ↓^***^ | 0.38 |
| Q8CGN5 | Plin1 | Perilipin-1 | | ↑^###^ | 2.86 | ↓^***^ | 0.34 |
| Q8C196 | Cps1 | Carbamoyl-phosphate synthase [ammonia], mitochondrial | | ↑^###^ | 2.09 | ↓^***^ | 0.38 |
| Q8BM72 | Hspa13 | Heat shock 70 kDa protein 13 | | ↑^###^ | 1.77 | ↓^***^ | 0.58 |
| Q8BJ34 | Marf1 | Meiosis regulator and mRNA stability factor 1 | | ↑^#^ | 1.83 | ↓^*^ | 0.61 |
| Q8BI29 | Sarg | Specifically androgen-regulated gene protein | | ↓^###^ | 0.50 | ↑^**^ | 1.57 |
| Q7TQ48 | Srl | Sarcalumenin | | ↑^###^ | 11.05 | ↓^***^ | 0.29 |
| Q792Z1 | Try10 | Trypsin 10 | | ↑^##^ | 1.76 | ↓^**^ | 0.47 |
| Q792Z0 | Prss3 | Protease, serine 3 | | ↑^###^ | 1.74 | ↓^***^ | 0.24 |
| Q6Q2Z6 | Acot5 | Acyl-coenzyme A thioesterase 5 | | ↓^###^ | 0.32 | ↑^*^ | 1.81 |
| Q6NV83 | U2surp | U2 snRNP-associated SURP motif-containing protein | | ↓^##^ | 0.48 | ↑^**^ | 1.59 |
| Q69ZP3 | Pnkd | Probable hydrolase PNKD | | ↓^#^ | 0.54 | ↑^**^ | 2.15 |
| Q64339 | Isg15 | Ubiquitin-like protein ISG15 | | ↓^###^ | 0.38 | ↑^***^ | 2.78 |
| Q64282 | Ifit1 | Interferon-induced protein with tetratricopeptide repeats 1 | | ↓^###^ | 0.14 | ↑^***^ | 8.13 |
| Q62264 | Thrsp | Thyroid hormone-inducible hepatic protein | | ↑^###^ | 2.55 | ↓^***^ | 0.65 |
| Q61176 | Arg1 | Arginase-1 | | ↑^##^ | 1.89 | ↓^*^ | 0.61 |
| Q60766 | Irgm1 | Immunity-related GTPase family M protein 1 | | ↓^###^ | 0.56 | ↑^***^ | 1.86 |
| Q5SX40 | Myh1 | Myosin-1 | | ↑^###^ | 3.09 | ↓^***^ | 0.43 |
| Q504N0 | Cpa2 | Carboxypeptidase A2 | | ↑^###^ | 1.61 | ↓^***^ | 0.30 |
| Q3USB7 | Plcl1 | Inactive phospholipase C-like protein 1 | | ↑^###^ | 2.79 | ↓^***^ | 0.41 |
| Q3UJU9 | Rmdn3 | Regulator of microtubule dynamics protein 3 | | ↑^#^ | 4.66 | ↓^*^ | 0.33 |
| Q3UIR3 | Dtx3l | E3 ubiquitin-protein ligase DTX3L | | ↓^#^ | 0.13 | ↑^***^ | 10.65 |
| Q3U5Q7 | Cmpk2 | UMP-CMP kinase 2, mitochondrial | | ↓^##^ | 0.52 | ↑^***^ | 2.49 |
| Q19LI2 | A1bg | Alpha-1B-glycoprotein | | ↑^###^ | 14.89 | ↓^***^ | 0.64 |
| Q14BI5 | Myom2 | Myomesin 2 | | ↑^###^ | 12.57 | ↓^**^ | 0.50 |
| Q08731 | Reg2 | Lithostathine-2 | | ↑^###^ | 6.78 | ↓^***^ | 0.34 |
| Q03963 | Eif2ak2 | Interferon-induced, double-stranded RNA-activated protein kinase | | ↓^###^ | 0.37 | ↑^**^ | 3.06 |
| P97457 | Mylpf | Myosin regulatory light chain 2, skeletal muscle isoform | | ↑^###^ | 3.12 | ↓^***^ | 0.45 |
| P50462 | Csrp3 | Cysteine and glycine-rich protein 3 | | ↑^###^ | 2.66 | ↓^***^ | 0.41 |
| P43137 | Reg1 | Lithostathine-1 | | ↑^###^ | 1.90 | ↓*** | 0.15 |
| P20801 | Tnnc2 | Troponin C, skeletal muscle | | ↑^###^ | 4.16 | ↓*** | 0.43 |
| P16015 | Ca3 | Carbonic anhydrase 3 | | ↑^###^ | 2.21 | ↓*** | 0.67 |
| P15947 | Klk1 | Kallikrein-1 | | ↑^###^ | 1.93 | ↓*** | 0.23 |
| P15089 | Cpa3 | Mast cell carboxypeptidase A | | ↓^###^ | 0.20 | ↑^*^ | 1.53 |
| P14427 | H2-D1 | H-2 class I histocompatibility antigen, D-P alpha chain | | ↑^###^ | 7.09 | ↓*** | 0.11 |
| P13542 | Myh8 | Myosin-8 | | ↑^###^ | 3.73 | ↓*** | 0.42 |
| P13541 | Myh3 | Myosin-3 | | ↑^###^ | 4.44 | ↓*** | 0.41 |
| P11928 | Oas1a | 2'-5'-oligoadenylate synthase 1A | | ↓^###^ | 0.59 | ↑^***^ | 1.85 |
| P07310 | Ckm | Creatine kinase M-type | | ↑^###^ | 2.07 | ↓*** | 0.58 |
| P07146 | Prss2 | Anionic trypsin-2 | | ↑^###^ | 1.51 | ↓*** | 0.32 |
| P05977 | Myl1 | Myosin light chain 1/3, skeletal muscle isoform | | ↑^###^ | 3.64 | ↓*** | 0.39 |
| P05208 | Cela2a | Chymotrypsin-like elastase family member 2A | | ↑^###^ | 1.55 | ↓*** | 0.36 |
| P04247 | Mb | Myoglobin | | ↑^###^ | 3.37 | ↓*** | 0.38 |
| P02816 | Pip | Prolactin-inducible protein homolog | | ↑^##^ | 2.41 | ↓^*^ | 0.56 |
| O70555 | Sprr2d | Small proline-rich protein 2D | | ↓^##^ | 0.47 | ↑^*^ | 1.65 |
| O54891 | Lgals6 | Galectin-6 | | ↑^###^ | 11.51 | ↓^***^ | 0.35 |
| O09165 | Casq1 | Calsequestrin-1 | | ↑^###^ | 4.92 | ↓*** | 0.25 |
| J3QNW0 | Dnmt1 | DNA （cytosine-5）-methyltransferase | | ↓^##^ | 0.39 | ↑^***^ | 2.90 |
| H3BJ30 | Cpsf6 | Cleavage and polyadenylation specificity factor subunit 6 | | ↓^##^ | 0.47 | ↑^***^ | 2.19 |
| G3UW82 | Myh2 | Myosin, heavy polypeptide 2, skeletal muscle, adult | | ↑^###^ | 3.52 | ↓^***^ | 0.48 |
| F7CVJ5 | Ahnak2 | AHNAK nucleoprotein 2 | | ↑^###^ | 1.54 | ↓^***^ | 0.39 |
| F6TLV3 | Fndc3a | Fibronectin type-III domain-containing protein 3A | | ↓^##^ | 0.56 | ↑^*^ | 1.59 |
| F2Z455 | Fhl3 | Four and a half LIM domains protein 3 | | ↑^###^ | 2.48 | ↓^***^ | 0.63 |
| Q9DCC4 | Pycr3 | Pyrroline-5-carboxylate reductase 3 | | ↓^##^ | 0.39 | ↑^***^ | 2.51 |
| E9Q9A9 | Oas2 | 2'-5'-oligoadenylate synthase 2 | | ↓^##^ | 0.33 | ↑^**^ | 2.47 |
| E9Q8K5 | Ttn | Titin | | ↑^###^ | 2.53 | ↓^***^ | 0.49 |
| E9Q1B2 | Tlcd3a | TLC domain-containing protein 3A | | ↓^##^ | 0.38 | ↑^*^ | 2.01 |
| E9PZQ1 | Ddx60 | DExD/H box helicase 60 | | ↓^##^ | 0.16 | ↑^**^ | 11.21 |
| E9PYJ9 | Ldb3 | LIM domain-binding protein 3 | | ↑^###^ | 4.44 | ↓^***^ | 0.23 |
| E9PYC2 | Dcpp2 | Demilune cell and parotid protein 2 | | ↑^###^ | 2.23 | ↓^***^ | 0.55 |
| E9PXF7 | Ifit1bl1 | Interferon-induced protein with tetratricpeptide repeats 1B-like 1 | | ↓^###^ | 0.20 | ↑^***^ | 5.37 |
| P70303 | Ctps2 | CTP synthase 2 | | ↑^###^ | 2.39 | ↓^***^ | 0.40 |
| E9PWK0 | Slc44a4 | Choline transporter-like protein 4 | | ↑^#^ | 1.80 | ↓^*^ | 0.60 |
| E9PWG4 | Myl1 | Myosin light chain 1/3, skeletal muscle isoform | | ↑^###^ | 4.39 | ↓^***^ | 0.29 |
| B2RU69 | Cuzd1 | CUB and zona pellucida-like domain-containing protein 1 | | ↑^###^ | 1.76 | ↓^***^ | 0.35 |
| B2RS76 | Cpb1 | carboxypeptidase B1 （Tissue） | | ↑^###^ | 1.54 | ↓^***^ | 0.34 |
| B1AR69 | Myh13 | Myosin, heavy polypeptide 13, skeletal muscle | | ↑^###^ | 2.97 | ↓^***^ | 0.42 |
| A3QM89 | Rtn1 | Reticulon | | ↑^#^ | 1.73 | ↓^*^ | 0.58 |
| A2RSF9 | Serpinb3c | Serine （Or cysteine） peptidase inhibitor, clade B, member 3C | | ↑^##^ | 2.35 | ↓^**^ | 0.51 |
| A2AUC9 | Klhl41 | Kelch-like protein 41 | | ↑^###^ | 6.53 | ↓^***^ | 0.26 |
| A2AR02 | Ppig | Peptidyl-prolyl cis-trans isomerase G | | ↓^##^ | 0.54 | ↑^**^ | 2.05 |
| A2AMY5 | Ubap2 | Ubiquitin-associated protein 2 | | ↓^###^ | 0.52 | ↑^*^ | 1.51 |
| A2AKH7 | Lrrc57 | Leucine-rich repeat-containing protein 57 | | ↓^#^ | 0.46 | ↑^*^ | 1.99 |
| A2AJW3 | Sycp2 | Synaptonemal complex protein 2 | | ↑^###^ | 3.44 | ↓^***^ | 0.36 |
| A2AJD1 | Bpifb9b | BPI fold-containing family B, member 9B | | ↑^##^ | 1.91 | ↓^**^ | 0.50 |
| A2A7S8 | Kiaa1522 | Uncharacterized protein KIAA1522 | | ↓^##^ | 0.42 | ↑^***^ | 2.64 |
| A2A6Q8 | Myl4 | Myosin light chain 4 | | ↑^###^ | 16.74 | ↓^***^ | 0.15 |
| A2A6K0 | Tnni2 | Troponin I, fast skeletal muscle | ↑^#^ | | 5.01 | ↓^*^ | 0.28 |
| A0A571BEN1 | Mybpc1 | Myosin-binding protein C, slow-type | ↑^###^ | | 2.91 | ↓^***^ | 0.62 |
| A0A1Y7VMH3 | Vrk1 | Serine/threonine-protein kinase VRK1 | ↓^##^ | | 0.21 | ↑^***^ | 6.61 |
| A0A1Y7VLZ7 | Pacs2 | Phosphofurin acidic cluster sorting protein 2 | ↓^#^ | | 0.42 | ↑^**^ | 3.03 |
| A0A1I7Q4G8 | Gatad2a | Transcriptional repressor p66 alpha | ↓^#^ | | 0.50 | ↑^*^ | 2.00 |
| A0A1B0GX24 | Sp100 | Nuclear autoantigen Sp-100 | ↓^###^ | | 0.30 | ↑^**^ | 2.44 |
| A0A140LI59 | Dmbt1 | Deleted in malignant brain tumors 1 protein | ↑^###^ | | 1.99 | ↓^***^ | 0.65 |
| A0A0R4J1B1 | Tnnt3 | Troponin T, fast skeletal muscle | ↑^###^ | | 2.67 | ↓^***^ | 0.47 |
| A0A0R4J1A8 | Tnnt3 | Troponin T, fast skeletal muscle | ↑^###^ | | 2.99 | ↓^***^ | 0.48 |
| A0A0R4J0I1 | Serpina3k | Serine protease inhibitor A3K | ↓^###^ | | 0.64 | ↑^***^ | 1.55 |
| A0A0G2JEA9 | Rbm8a | RNA-binding protein 8A | ↓^#^ | | 0.66 | ↑^**^ | 1.60 |
| A0A0G2JDW2 | Myl3 | Myosin light chain 3 | ↑^###^ | | 3.43 | ↓^***^ | 0.48 |
| A0A075B5P6 | Ighm | Immunoglobulin heavy constant mu | ↓^###^ | | 0.40 | ↑^**^ | 1.73 |

^#^*p*<0.05, ^##^p<0.01, ^###^*p*<0.001 compared with control; ^*^*p*<0.05， ^**^*p*<0.01，^***^*p*<0.001 compared with model; “↑”, increase in signal; “↓”, decrease in signal; Data are expressed as mean ±S.D（*n* = 5）.
